# Bibliometric and Visual Analysis of Global Research on Taurine, Creatine, Carnosine, and Anserine with Metabolic Syndrome: From 1992 to 2022

**DOI:** 10.3390/nu15153374

**Published:** 2023-07-29

**Authors:** Jiaru Sun, Fang Guo, Jinjun Ran, Haisheng Wu, Yang Li, Mingxu Wang, Xiaoqin Wang

**Affiliations:** 1 Department of Nursing, Xi’an Jiaotong University Health Science Center, 76 Yanta West Road, Xi’an 710061, China; r17691212164@163.com; 2 School of Public Health, The University of Hong Kong, 7 Sassoon Road, Pok Fu Lam, Hong Kong, China; haisheng@connect.hku.hk (H.W.); yangli-01@connect.hku.hk (Y.L.); 3 School of Public Health, Shanghai Jiao Tong University School of Medicine, Shanghai 200092, China; jinjunr@sjtu.edu.cn; 4 School of Public Health, Xi’an Jiaotong University Health Science Center, 76 Yanta West Road, Xi’an 710061, China

**Keywords:** taurine, creatine, carnosine, anserine, metabolic syndrome, bibliometric analysis

## Abstract

Red meat and animal-sourced protein are often disparaged as risk factors for developing metabolic syndrome, while emerging research has shown the beneficial effects of dietary taurine, creatine, carnosine, and anserine which are all exclusively abundant in red meat. Thus, it is imperative to highlight the available evidence to help promote red meat as part of a well-balanced diet to optimize human health. In this study, a bibliometric analysis was conducted to investigate the current research status of dietary taurine, creatine, carnosine, and anserine with metabolic syndrome, identify research hotspots, and delineate developmental trends by utilizing the visualization software CiteSpace. A total of 1094 publications were retrieved via the Web of Science Core Collection from 1992 to 2022. There exists a gradual increase in the number of publications on this topic, but there is still much room for research papers to rise. The United States has participated in the most studies, followed by China and Japan. The University of Sao Paulo was the research institute contributing the most; Kyung Ja Chang and Sanya Roysommuti have been identified as the most prolific authors. The analysis of keywords reveals that obesity, lipid profiles, blood pressure, and glucose metabolism, as well as ergogenic aid and growth promoter have been the research hotspots. Inflammation and diabetic nephropathy will likely be frontiers of future research related to dietary taurine, creatine, carnosine, and anserine. Overall, this paper may provide insights for researchers to further delve into this field and enlist the greater community to re-evaluate the health effects of red meat.

## 1. Introduction

Metabolic syndrome (MetS), characterized by a cluster of metabolic disorders (increased blood pressure, impaired fasting blood glucose, abnormal cholesterol and/or triglyceride [TG] levels, and abdominal obesity), is a serious pathologic condition tied to greatly raised risks of cardiovascular and cerebrovascular diseases and so is a heavy public health burden globally [1,2]. It is estimated that about one out of three adults will contract MetS, and the prevalence rate is increasing especially among the young [3]. Under such context, tremendous efforts have been made to investigate the modifiable risk factors for MetS, among which diet is a critical component and hot research spot.

In recent decades, there have been mounting studies reporting an observational link between red meat (e.g., beef) intake and a multitude of chronic health conditions including MetS [4], which has led several institutions such as the WHO to recommend limiting the consumption of red meat but with nonuniform targets [5,6,7,8]. Recently, Murray et al. who scrutinized decades of relevant research has slammed such an assertion, since they summarized very weak evidence of health risks related to unprocessed red meat [9]. Adding to this ambiguity, scientific debate surrounding the nutritional advantage of animal- versus plant-sourced protein remains lingering, but the lay public is often informed to substitute the former with the latter for health concerns.

Meanwhile, emerging research reveals that taurine, creatine, carnosine, and anserine, which all abound in red meat, are nutritionally important to physical health by attenuating oxidative stress and inflammation, and so for promoting metabolic profiles [10]. For example, taurine is a functional amino acid essential for children’s growth and conditionally essential for adults to optimize health. It can exert cardiovascular and metabolic protection effects by maintaining the integrity of cell membranes, lowering blood pressure, and limiting ischemia-reperfusion injury [11,12,13]. Carnosine and anserine are dipeptides with physiological significance in ameliorating the development of MetS due to their antioxidant capacity, anti-lipidemic, and anti-glycation actions [14,15]. Creatine, a metabolite of amino acids, is found to involve antioxidative and anti-apoptotic reactions, and modulate energy metabolism in the excitable tissues (i.e., brain and skeletal muscle) [16,17]. Of note, all these functional nutrients, which are particularly plentiful in beef, are absent from plant-sourced foods including cereals (e.g., wheat flour, white rice, and corn grains), tubers (e.g., potatoes and sweet potatoes), legumes (e.g., soybeans), and nuts (e.g., peanuts, pistachio nuts) [18,19,20]. Therefore, it is imperative to highlight the relevant research findings, so as to help improve the understanding of red meat as an irreplaceable part of a well-balanced diet to optimize human health. Although qualitative review has summarized the physiology and health benefits of taurine, creatine, carnosine, and anserine [10], bibliometric method has yet not been applied to quantitatively depict the research status concerning the relationships of those physiologically important nutrients with MetS [21].

At this juncture, it would be necessary to distinguish bibliometric analysis from frequently used literature reviews. While the latter relies on qualitative research procedures (e.g., thematic analyses) done manually by scholars to acquire and assess the extant literature of a well-defined research area, bibliometric analysis encapsulates quantitative techniques applied to massive bibliometric data via developed algorithms to summarize the intellectual structure and development of a research domain [21]. Through analyzing the networks and characteristics of multilevel research constituents (e.g., topics, citations, etc.), scholars could objectively evaluate the literature and decipher research hotspots as well as evolving trends.

This is where our work enters the picture. A bibliometric analysis is performed to map and decipher the cumulative scientific knowledge in the research domain associating dietary taurine, creatine, carnosine, and anserine with MetS, delineate its evolution process, and explore research hotspots and frontiers. Enhancing understanding of relevant scientific knowledge by bibliometrics can facilitate research incubation and academic development.

## 2. Methods

### 2.1. Data Collection

The literature was extracted from Web of Science Core Collection (WoSCC) on 2 January 2022. The search formula was as follows: TS = ((Mets) OR (metabolic syndrome) OR (obesity) OR (overweight) OR (triglycerides) OR (HDL-c) OR (cholesterol*) OR (hypertension) OR (blood pressure) OR (hyperglycemia) OR (hyperglycaemia) OR (diabetes) [Topic]) AND ((taurine) OR (carnosine) OR (anserine) OR (creatine)[Title]). The retrieval time span was from 1992 to 2022, the document type was selected as “article” or “review”, and the publication language was limited to English. The literature screening process was conducted independently by two researchers, and divergent opinions would be determined through discussions or a third researcher. We eliminated 566 publications, including the following types: (a) papers inconsistent with the topic of taurine, creatine, carnosine, and anserine with MetS (*n* = 553); (b) CiteSpace software failed to identify the title/country/institution/author/keyword/reference of the papers (*n* = 11); and duplicate papers (*n* = 2). Finally, a total of 1094 papers were obtained and exported in the form of “Full Record and Cited References” (Figure S1).

### 2.2. Data Analysis

CiteSpace software is a visualization analysis tool combining data mining algorithms, information visualization, and bibliometrics [22]. It intuitively maps the hotspots and evolution processes of a certain research field, as well as predicting its frontiers and development trends. This paper applied the 6.2.R3, 64-bit version of CiteSpace software to perform annual publication analysis, journal distribution and representative literature analysis, collaboration analysis, keyword cluster analysis, and burst keyword analysis of the included literature. We set the parameters of the CiteSpace as follows: (a) timespan = 1992–2022, year per slice = 1; (b) term source = title/abstract/author keywords/keywords plus; (c) node types = country/institution/author/keyword/reference; (d) threshold selection criteria = the top 50 items for each time slice; we defaulted to the settings for the other parameters.

The following displays a brief introduction of the main analyses (please see the details of each analysis in the supplementary file and figure legends): Collaboration analysis was employed to examine the contribution of countries/regions, institutions, and authors, as well as their in-between cooperation. The visualized maps consist of two important elements, node (whose size represents the number of published papers) and link (whose number and thickness represent the collaboration relationship). The thickness of purple ring represents the centrality (i.e., the influence and intermediary connection degree) strength of nodes in the knowledge networks. Centrality > 0.1 indicates that the node is a central node with stronger research influence [23]. Keyword cluster analysis integrates synonyms into the same cluster to recognize buzz keywords and thus research hotspots in the field. Modularity Q > 0.3 indicates that there is a significant cluster structure; a mean Silhouette score > 0.5 suggests that the clustering results are robust [23]. Burst keyword analysis was conducted to detect the keywords with large frequency change and fast growth rate in a short period, in order to explore the trends and dynamic frontiers of research hotspots.

## 3. Results

### 3.1. Annual Publications

**Figure 1:**
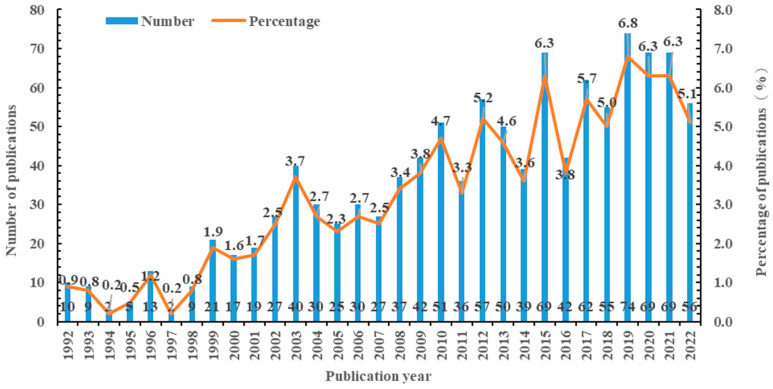
The annual number of publications on taurine, carnosine, anserine, and creatine with metabolic syndrome from 1992 to 2022.

A total of 1094 related publications were retrieved via WoSCC from 1992 to 2022. Despite a slight fluctuation, the number of publications over the past 30 years has shown an overall upward trend (Figure 1). From 1992 to 2001, the research of taurine, carnosine, anserine, and creatine in association with MetS were still in the embryonic stage, and the output of publications was low, with an average annual publication of only 10.7. From 2002 to 2011, the number of publications has steadily increased and was 3.2 times higher than the previous decade. From 2012 to 2022, the growth rate of publications has increased slightly, culminating in 2019 with 74. During this period, the number of publications accounted for 58.7% of the total. Obviously, there is still room for the number of publications to rise The relationship of taurine, carnosine, anserine, and creatine with MetS is still a hot field with potential.

### 3.2. Distribution of Journals and Representative Literature

We found that 1094 papers regarding taurine, carnosine, anserine, and creatine in association with MetS were published in 447 academic journals. The top 12 journals with the largest output contributed 19.10% of the total publications, among which the journal of *Amino Acids* was far ahead (*n* = 73, 6.67%), followed by *Aquaculture* (*n* = 17, 1.55%) (Table S1)*. Journal of Biomedical Science*, ranking No. 5, had the highest impact factors (IF) of 12.771. Of note, approximately 67% of the top 12 journals were in the Q1 region. 

This paper also summarized 10 representative references in this research field (Table S2). About 60% of the top 10 references were published in Q1 journals, of which the highest IF was up to 46.500 (*Physiol Rev*). The research group of B. de Courten, et al. [24] has been cited the most and has the highest centrality, reflecting the high contribution and recognition of this paper in this field. It is a double-blind randomized pilot trial to test the effect of carnosine supplementation on glucose metabolism. Then, six references were review articles [14,25,26,27,28,29] and the other three references were mice-based basic research, including the role of taurine supplementation on glucose homeostasis and islet function [30], the treatment effect of carnosine on diabetes and diabetic nephropathy [31], and the mechanisms of carnosine ameliorating dyslipidemia, hypertension, and renal function [32]. 

### 3.3. Collaboration Analysis

**Figure 2:**
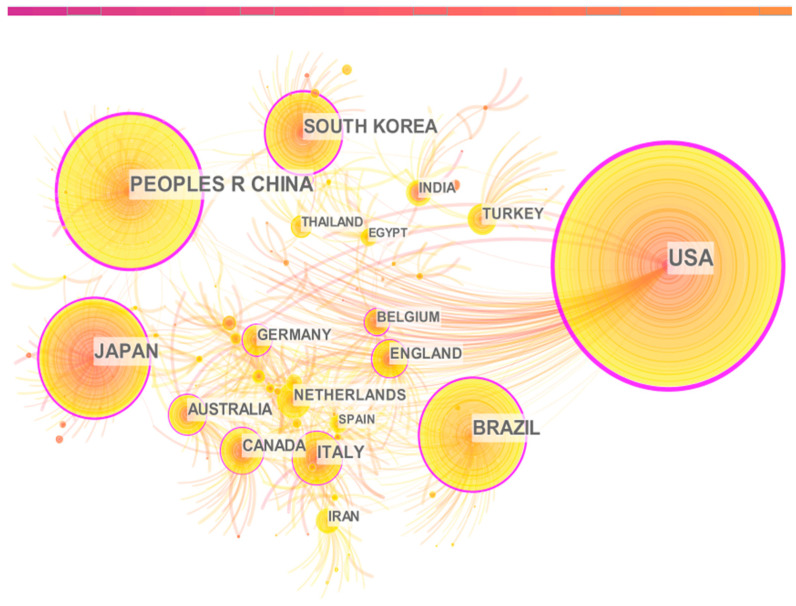
Collaboration network among research units at the countries/regions level in the research field of taurine, creatine, carnosine, and anserine with metabolic syndrome. Here, research constituents of larger node size reveal that more papers have been published from the unit. Nodes with purple ring indicate that they are central nodes in the network with the scale of centrality > 0.1 (i.e., representing stronger influence and more intermediary connection in the knowledge networks). The thickness of the purple ring is proportional to the centrality strength of the nodes (i.e., the higher the centrality, the greater the influence of the research unit, the closer the connection with others). The number and thickness of links between nodes reflect their collaboration relationships. The color of nodes and links represent publication and first association time, respectively. Warmer color indicates a closer time.

The collaboration network of countries/regions was generated to identify their scientific research contribution and cooperation relationships in this field (Figure 2). The collaboration map consists of 866 nodes and 1052 lines, with a network density of 0.0028. The USA contributed the largest number of publications (*n* = 207, 18.92%), followed by China (*n* = 131, 11.97%) and Japan (*n* = 101, 9.23%). Those top three countries published 439 papers on the topic of interest, accounting for 40.13% of the whole publication pool (Table S3). High production countries/regions showed active cooperation, with the top 10 countries/regions centrality all above 0.1, except for the Netherlands. The influence of the USA was the most prominent with a centrality of 0.79, with China (0.53) and Brazil (0.27) ranking the second and the third, respectively. However, the cooperation among the rest of the countries/regions, especially low production ones, was scattered and lacking stability.

The institution-level collaboration network was also mapped to explore influential institutions and analyze their collaboration degree (Figure S2). The network is chaotic, containing 1452 nodes and 2620 links with a network density of 0.0025. Up to 90% of the top 10 institutions regarding output were universities (Table S3). The University of Sao Paulo has contributed the most (*n* = 38, 3.47%), followed by Inha University (*n* = 33, 3.02%), and Khon Kaen University (*n* = 21, 1.92%). The University of Milan (0.18) and the University of Sao Paulo (0.10) have shown a high centrality, acting as bridges between institutional cooperation. 

By selecting the “author” node type to analyze the core authors and their collaboration in this research topic (Figure S3), the yielded network contains 4527 nodes and 15,102 links, with a network density of 0.0015. The top 10 authors all published more than 10 papers (Table S3). Among them, Kyung Ja Chang and Sanya Roysommuti contributed the largest number of publications (*n* = 21, 1.92%), followed by Bruno Gualano (*n* = 19, 1.74%). Although the collaboration network among authors presents some obvious team cooperation relationships, the overall cooperation hitherto is still insufficient (centrality was all less than 0.01).

### 3.4. Keyword Cluster Analysis

**Figure 3:**
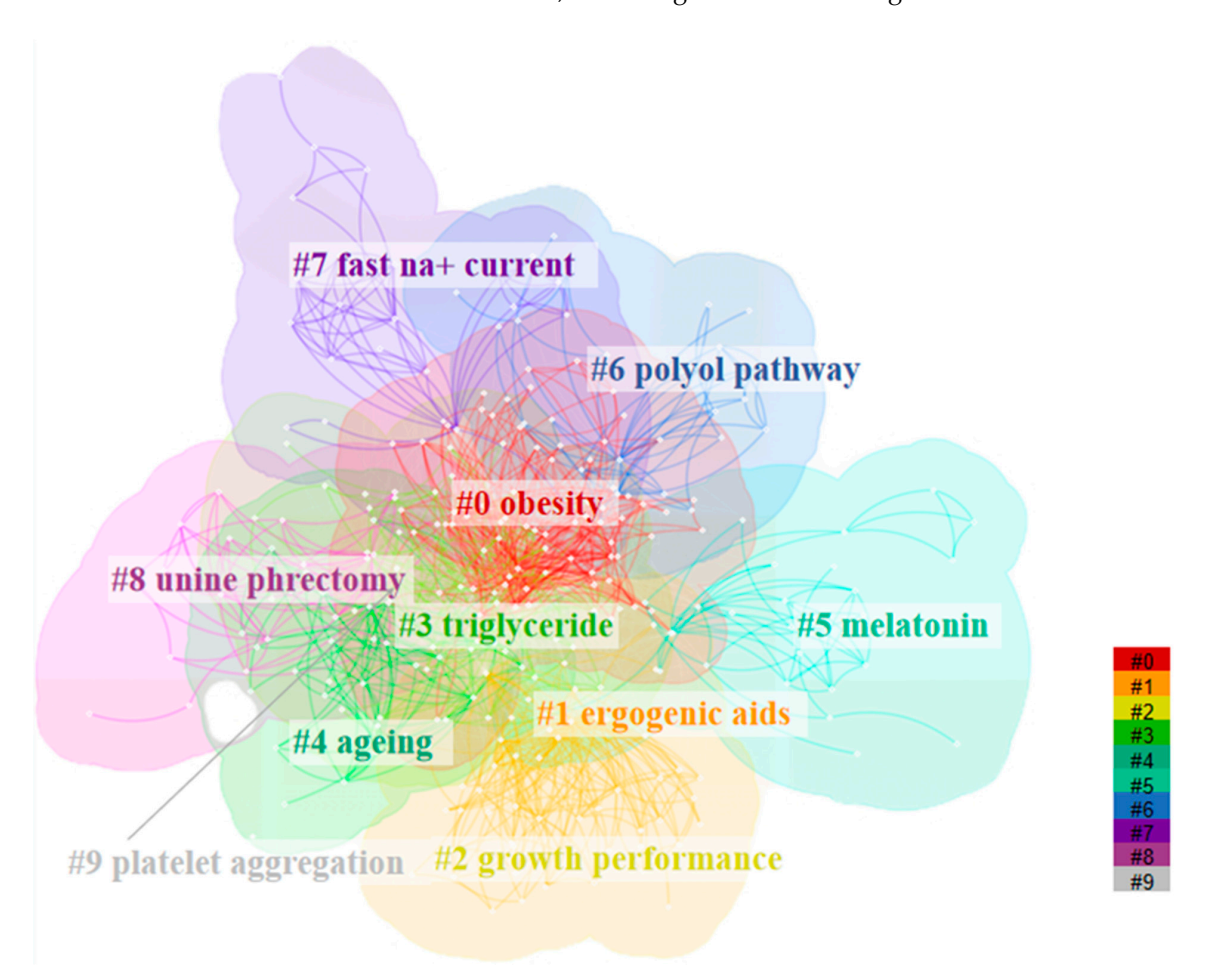
Cluster map of keywords on taurine, carnosine, anserine, and creatine with metabolic syndrome. Different colors were used to distinguish between the different clusters. The node (small white dot) represents each publication. And the publications with the same keyword (including its synonyms) are linked by edges, of which the color is specifically defined for each cluster.

Keyword cluster analysis was performed to illustrate the hotspots of research on taurine, carnosine, anserine, and creatine with MetS (Figure 3). The network of keywords cluster consists of 1375 nodes and 9063 lines, with a network density of 0.0096. The most frequent keywords of studies were “taurine”, “oxidative stress”, “metabolism”, “rat”, “supplementation”, etc. (Table 1). The centrality of keywords was all less than 0.1, with “cholesterol”, “blood pressure”, “insulin”, and “kidney” being the top four. Furthermore, the keywords were divided into 10 clusters, including “obesity”, “ergogenic aids”, “growth performance”, “triglyceride”, “aging”, “melatonin”, “polyol pathway”, “fast na+ current”, “unine phrectomy”, and “platelet aggregation”. The modularity Q was 0.549, and the mean Silhouette score was 0.504, indicating that the clustering was reasonable.

### 3.5. Burst Keyword Analysis

**Figure 4:**
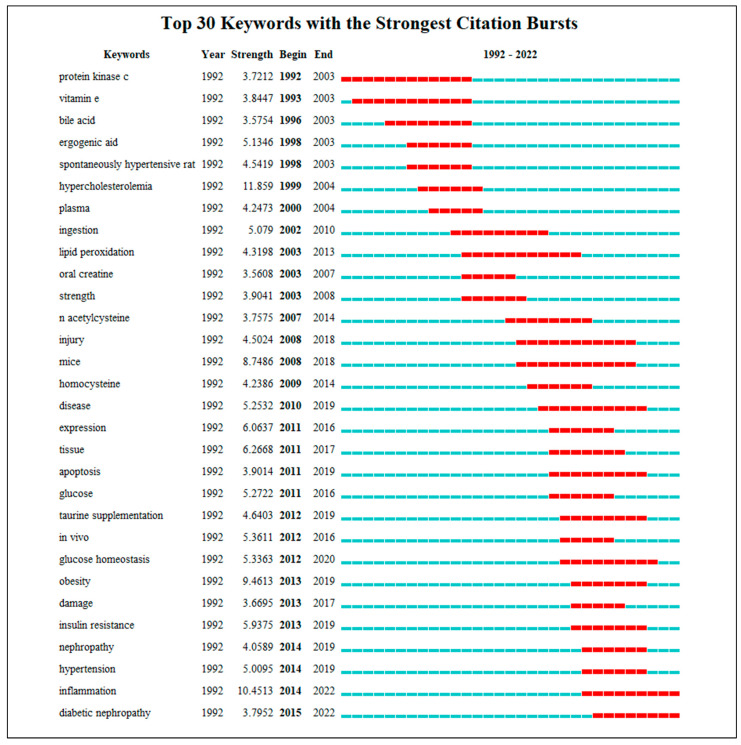
Top 30 keywords on taurine, carnosine, anserine, and creatine with metabolic syndrome with the strongest citation bursts from 1992 to 2022. This figure emphasizes the burst time and strength of the keywords. “Year” indicates the year when the keyword first appeared, while “Begin” and “End” represent the start and end year of the keyword as a hotspot. Each segment on the line represents a year, and the active period of keywords with the strongest citation bursts has been marked in red, indicative of a research frontier for that stage.

Burst keyword analysis reveals large changes in the keywords in a short period, in order to detect the frontier and dynamic trends in the field (Figure 4). This study identified the top 30 keywords with high burst strength (>3.5, indicating that the keyword appears more frequently during the detected period) in the research field focusing on taurine, carnosine, anserine, and creatine in association with MetS. The strongest burst keyword was “hypercholesterolemia”, with a burst strength reaching 11.859. About burst time, 53% of the keywords appeared with bursts between 2014 and 2015. The keyword with the longest burst time was “protein kinase c”, lasting 10 years. The burst times of “inflammation” and “diabetic nephropathy” have continued today and may remain as hot keywords for future research.

## 4. Discussion

### 4.1. General Summary

By conducting a bibliometric analysis of literature regarding the association of dietary taurine, creatine, carnosine, and anserine with MetS over the last 30 years, maps and tables are generated to help decipher the research status uncovering hotspots and emerging trends, as well as to explore the intellectual structure of this domain intuitively. In general, the past three decades have seen a gradual increase in the numbers of publications on this topic, reaching a peak in 2019. Yet, more concerted endeavor is pressingly needed given the current misconception of red meat’s nutritive value. The United States has published the most articles, followed by China and Japan. The top three institutions for the number of publications are the University of Sao Paulo, Inha University, and Khon Kaen University. Kyung Ja Chang and Sanya Roysommuti are identified as the most prolific authors in this field, followed by Bruno Gualano. Given the inconsistent patterns between active research constituents (e.g., countries, institutions, authors), it can be inferred that this research area remains to further flourish and the collaboration degree as well as the research impact is not sufficient.

### 4.2. Research Hotspots

The map of keywords co-occurrence based on algorithms deciphers that literature of dietary taurine, carnosine, anserine, and creatine with MetS shines through ten clusters. Taking also frequency and centrality (i.e., link strength) of representative keywords into account, we summarized research hotspots in this field as follows. 

Firstly, obesity is a clinical outcome of particular concern when examining the health effects of dietary functional amino acids, especially taurine [33,34,35]. Various animal models have shown the anti-obesity effects of taurine supplementation, tied to decreased body weight with inhibited expression of adipogenic genes but improved expression of hepatic genes involved in fatty acid metabolism [36,37]. The underlying pathways may include lower food/caloric intake via preserving hypothalamic leptin action and modulating circadian rhythms in high-fat diet-induced obese mice [38,39]. Moreover, taurine may also modulate energy metabolism to attenuate or prevent obesity [35]. An increase in resting oxygen expenditure was found in obese mice supplemented with taurine, and it may be ascribed to upregulated expression of energy expenditure related genes such as peroxisome proliferator-activated receptors (PPARs) and uncoupling protein (UCP) in white adipose tissue, as well as an increase in gene expression during adipocyte browning and fatty acid oxidation such as UCP1, mitochondrial cytochrome c (Cyc), etc. [35]. Up to now, the anti-obesity effect of taurine and its underlying mechanisms are much more studied in animal models than in humans [40,41]; thus, more population-based studies are strongly warranted to evidence the role of taurine and other dipeptides in metabolic health outcomes including obesity.

On the other hand, animal models and subpopulations with obesity or diabetes are often targeted to explore the health effects of dietary taurine and carnosine on plasma lipid profiles (such as TG and cholesterol content), blood pressure, and glucose metabolism [34,42,43,44,45]. Taurine administration (as a small proportion in diet/drinking water) for several weeks could significantly reduce serum TG, cholesterol, and low-density lipoprotein cholesterol (LDL-C) levels in animals [46,47]. Such hypolipidemic effect has also been documented by a limited number of randomized trials among human populations [40,42,45,48]. Amelioration of dyslipidemia status has been assumed to relate to improved insulin sensitivity and leptin modulation, as well as obesity prevention [34,49]. Regarding blood pressure, rats with taurine deficiency contracted more severe hypertension than their control counterparts, and elevated blood pressure could be reduced by taurine treatment [50,51], potentially through modulating angiotensin-converting enzyme (ACE) activity and nitric oxide (NO) synthesis [51,52]. Negative associations between 24-hour urinary taurine excretion and blood pressure in humans were also reported [53,54]. As for glucose metabolism, animals or humans supplemented with taurine or carnosine or creatine showed significant attenuation in increased plasma glucose, glycated protein, and glycated hemoglobin levels induced by a glucose tolerance test or a high-fructose diet [55,56,57,58], possibly through reducing the expression of gluconeogenic genes while increasing the hepatic expression of glycolytic genes [59]. Alongside rectifying hepatic glucose metabolism, taurine or carnosine could enhance hepatic insulin signaling to improve glucose tolerance and compensate for insulin resistance [24,60,61]. In contrast, studies examining the health effects of anserine and creatine on obesity and its related conditions such as hyperlipemia and high blood pressure, as well as hyperglycemia, remain rather limited [62,63], so more research efforts are solicited.

In addition to the metabolic conditions of interest as health outcomes, another popular research focus is dietary creatine supplementation as nutritional ergogenic aid to promote greater gains in fat-free mass, muscular strength, agility, endurance, and sprint performance during intense exercise than is achievable with strength training or resistance training alone [64,65,66]. Apart from athletic improvement, researchers also showed that dietary creatine could contribute to injury prevention, thermoregulation, post-exercise recovery, and rehabilitation [67]. Poor body composition and declining physical function are highly prevalent with aging, so older adults are a subpopulation of special interest when examining the health effects of creatine supplementation. Interventional studies found that creatine supplementation combined with resistance or strength training could effectively improve lean tissue mass and muscle strength in aging adults [68,69,70,71]. Beyond that, dietary taurine supplementation flourishes in aquaculture and agricultural science. Many feeding experiments have shown that the addition of taurine to high-carbohydrate or plant-based diets could improve growth performance and lipid metabolism as well as antioxidant capacity in various juvenile fishes and broiler chickens [72,73,74,75]. It is suggested that randomized trials are also designed and performed in adolescents to examine the effect of taurine supplementation on growth rate and metabolic parameters, especially among those with plant-based diets [76].

Melatonin, an endogenous hormone related to circadian rhythms, has gained research interest in its potential role of improving glycemic homeostasis; therefore, it often serves as a control/comparative group in the study of taurine’s health effects in diabetic rats [13,75]. In the polyol pathway, glucose is reduced to sorbitol intracellularly and further oxidized to fructose; this pathway is suspected to be implicated in diabetic complications and associated microvascular damage as well as cellular dysfunctions, leading to retinopathy, nephropathy, neuropathy, platelet aggregation, and atherosclerosis [77]. Altered taurine metabolism has been found to be involved in a pathologic process related to the polyol pathway, and taurine dietary supplements were proposed to hold potential therapeutic perspectives in combination with other drugs [25]. Vast studies around the 1990s have focused on the role of taurine in modulating Ca^2+^ homeostasis; it was reported that taurine could inhibit the tetrodotoxin-sensitive fast Na^+^ channels, accompanied by increased intracellular Ca^2+^, leading to a positive cardiac inotropic effect [78]. Tissue taurine levels are also known to modulate renal function and blood pressure, so rat models with uninephrectomy have been used to test the protective effect of taurine treatment on kidneys, and the detrimental effect of taurine deficiency on accelerating hypertension development [50,79]. In addition to that, taurine and carnosine showed effectiveness in reducing oxidative stress and inhibiting platelet aggregation, thereby offering potentially greater cardiovascular protection [80,81]. Given the versatile properties of taurine and other functional dipeptides, it is imperative to validate existing findings, and yield new insights about their health benefits especially in humans, as well as the underlying mechanisms, so as to advocate for the necessity of including red meat in a daily diet.

### 4.3. Research Frontiers

The timeline view of keywords citation burst reveals the development trends and frontiers in the research field of dietary taurine, carnosine, anserine, and creatine with MetS. Before the year 2000, the study of taurine, carnosine, anserine, and creatine was at the beginning stage, devoted mainly to illustrating the regulation factors (e.g., protein kinase C), including its role in bile synthesis, membrane protection, ergogenic aid for exercise, and cholesterol metabolism from a broad angle [65,82,83]. In the next 10 years, the objects of studies have focused more on oral supplementation especially in mice models, and the health effects on not only muscle strength, but also injury and disease risks [84,85]. *N*-acetylcysteine and homocysteine were the two kinds of amino acids studied often in parallel with taurine [86,87]. After 2010, the research community began to explore the modulating effects of taurine supplementation on gene expressions (including apoptosis markers), as well as its role in preventing obesity, maintaining glucose homeostasis, and improving insulin resistance [26,88,89]. In the most recent years, the research focus became more refined. The functions of taurine supplements on ameliorating nephropathy and hypertension, as well as chronic inflammation as implicated in all the MetS conditions, have been intensively investigated [90,91,92]. Moreover, diabetic nephropathy as the most common complication in diabetes patients is gaining increasing research interest when examining the health effects of taurine and carnosine [93,94]. This highlights the research frontiers and future directions of this field. 

While a bunch of studies among various populations have investigated the relationship of red meat intake with the risk of colorectal cancer, breast cancer, and cardiovascular diseases, those findings are mixed [95,96,97]. The most recent systematic review and meta-regression published in *Nature Medicine* have concluded with a very weak association of unprocessed red meat with colorectal cancer but no significant association with either ischemic heart disease or stroke or diabetes [9]. It is a fact that previous population-based studies reporting detrimental health effects of red meat are almost of an observational nature; therefore, strengthened research evidence is pressingly warranted. Given the available interventional study findings among animals reporting beneficial effects of taurine, carnosine, anserine, and creatine on various metabolic conditions, we envision that randomized controlled trials among populations should be better supplemented to confirm their health effects, adding the reliability of promoting appropriate intake of red meat which is exclusively rich in those key nutrients.

### 4.4. Strengths and Limitations

This is the first bibliometric analysis to map and analyze the cumulative scientific knowledge of the research field regarding dietary taurine, carnosine, anserine, and creatine with MetS from large volumes of unstructured data. It can empower scholars in this area to obtain a one-stop overview, spot knowledge gaps, and formulate novel research ideas, thereby advancing academic development and better informing the public and relevant policymakers. But the limitations of this study should also be noted. Firstly, only the WoSCC database was retrieved for bibliometric analysis. Although it is deemed reliable and authoritative, the data would be more comprehensive if other database resources (e.g., Embase) could be added. Secondly, only English literature were extracted via specific search strings, so language-related bias cannot be avoided. However, it is nearly impossible to include all the relevant papers; scholars with research interests into specific topics are suggested to further delve into the literature exquisitely.

## 5. Conclusions

To sum up, the current study provides a quantitative and qualitative bibliometric analysis of literature in the field of dietary taurine, carnosine, anserine, and creatine with MetS. The research hotspots summarized include their health effects on obesity, lipid profiles, blood pressure, and glucose metabolism, as well as their functions as ergogenic aid and growth promoter. Animal models were utilized extensively, so population-based studies and randomized trials are necessary to expand and validate the current findings. Furthermore, enhanced cooperation among countries, institutions, and authors is needed for this research field to further prosper. Given the metabolic protection effects of taurine together with other functional dipeptides and their abundance in red meat, our research serves as a reference for more in-depth investigation and re-evaluation of the health effects of red meat on metabolic diseases.

## Figures and Tables

**Table 1:** Top 10 keywords on taurine, carnosine, anserine, and creatine with metabolic syndrome.

No.	Keywords	Frequency ^a^	Keywords	Centrality ^b^
1	Taurine	365	Cholesterol	0.09
2	Oxidative stress	167	Blood pressure	0.08
3	Metabolism	158	Insulin	0.07
4	Rat	156	Kidney	0.07
5	Supplementation	156	Expression	0.06
6	Skeletal Muscle	92	Damage	0.06
7	Glucose	91	Hyperglycemia	0.06
8	Exercise	90	Supplementation	0.05
9	Carnosine	88	Exercise	0.05
10	Expression	81	Diabetes	0.05

^a^: Frequency indicates the number of publications indexed with certain keywords. ^b^: The higher the centrality, the greater the influence, the closer the connection with others.

## Data Availability

No new data were created or analyzed in this study. Data sharing is not applicable to this article.

## Supplementary Materials

The following supporting information can be downloaded at: https://www.mdpi.com/article/10.3390/nu15153374/s1, Figure S1: Flowchart of literature selection; Figure S2: Collaboration network among research units at the institution level in the research field of taurine, creatine, carnosine, and anserine with metabolic syndrome; Figure S3: Collaboration network among research units at the authorship level in the research field of taurine, creatine, carnosine, and anserine with metabolic syndrome; Table S1: Top 12 most prolific journals related to taurine, carnosine, anserine and creatine with metabolic syndrome; Table S2: Top 10 cited references on taurine, carnosine, anserine and creatine with metabolic syndrome; Table S3: Top 10 countries/regions, institutions and authors of publications related to taurine, carnosine, anserine and creatine with metabolic syndrome.
