# Supplementary material for: Bibliometric and Visual Analysis of Global Research on Taurine, Creatine, Carnosine, and Anserine with Metabolic Syndrome: From 1992 to 2022
Source: Nutrients. 2023 Jul 29;15(15):3374. doi: 10.3390/nu15153374 (PMC10420945; doi:10.3390/nu15153374)
Supplement: Supplementary file 1 [file nutrients-15-03374-s001.zip › nutrients-2521168-supplementary.pdf]

Bibliometric and visual analysis of global research on taurine, creatine, carnosine, and anserine with metabolic syndrome: From 1992 to 2022

Jiaru Sun[1,#], Fang Guo[2,#,*], Jinjun Ran[3], Haisheng Wu[2], Yang Li[2], Mingxu Wang[4,*], Xiaoqin Wang[1,*]

[1]Department of Nursing, Xi'an Jiaotong University Health Science Center, Xi'an, China
[2]School of Public Health, The University of Hong Kong, Pok Fu Lam, Hong Kong, China
[3]School of Public Health, Shanghai Jiao Tong University School of Medicine, Shanghai, China
[4]School of Public Health, Xi'an Jiaotong University Health Science Center, Xi'an, China

*** Correspondence:**
Xiaoqin Wang
Address: Department of Nursing, Xi'an Jiaotong University Health Science Center, 76 Yanta West Road, Xi'an 710061, China
E-mail: wangxiaoqin@mail.xjtu.edu.cn;

Fang Guo
Address: School of Public Health, The University of Hong Kong, 7 Sassoon Road, Pok Fu Lam, Hong Kong, China
E-mail: guof0818@hku.hk;

Mingxu Wang
Address: School of Public Health, Xi'an Jiaotong University Health Science Center, 76 Yanta West
Road, Xi'an 710061, China
E-mail: wangmx601@mail.xjtu.edu.cn

# These two authors have contributed equally to this work.

Supplementary methods

This section serves as a detailed explanation of each bibliometric analysis conducted in this study.

a) The annual publication analysis was performed to show the annual publications on taurine, creatine, carnosine, and anserine with metabolic syndrome (MetS) in the past 30 years, which to an extent reflects the attention of scholars and dynamic trend in this field.
b) Journal distribution analysis presents the top 12 most prolific journals, as well as the journal publication number, percentage, impact factor (IF), and Journal Citation Reports cate-gory, which were used to identify the degree of development and influential journals in the field. This study also indexed the most cited literature references in this field.
c) Collaboration analysis was employed to examine the contribution of countries/regions, institutions, authors and the cooperation between them. The visualization knowledge maps consist of two important elements, node and link. The size of nodes represents the number of published papers. The number and thickness of connection links between nodes reflect the collaboration relationships. The colors of nodes and lines represent different years and the first association time, respectively. Warmer color indicates a closer time. The thickness of purple ring represents the centrality strength of nodes. Centrality implies the influence and intermediary connection degree of nodes in the knowledge networks. Centrality >0.1 indicates that the node is a central node, representing stronger influence in the research networks. Moreover, the higher the centrality, the greater the influence of the country/region, institution, and author, the closer the connection with the surrounding units (1). Besides, network density reveals the overall status of cooperation between nodes; density values range between 0 and 1, with lower density values indicating more dispersed cooperation.
d) Keyword cluster analysis integrates synonyms into the same cluster to recognize hot keywords in the field. Modularity Q >0.3 indicates that there is a significant cluster structure. A mean Silhouette score >0.5 suggests the robustness of clustering results, while Silhouette score >0.7 implies that the clustering results are significant (1).
e) Burst keyword analysis was conducted to detect the keywords with large frequency change and fast growth rate in a short period, to explore the trend and frontiers of research hotspots. It emphasizes the burst time and strength of the keyword. “Year” indicates the year when the keyword first appeared, “Begin” and “End” represent the start and end year of the keyword as a hotspot. Each line means a year, the red line represents the duration stage of the keyword as the frontier, and the blue represents that the keyword is not paying enough attention in the current time. “Strength” implies the burst strength of the keyword: the greater the value, the greater the burst strength, indicating that the more frequently the keyword appears during this period.

Reference

1. Chen Y. The Methodology Function of Cite Space Mapping Knowledge Domains. *Stud. Sci. Sci.* **2015**; 33: 242-253. doi:10.16192/j.cnki.1003-2053.2015.02.009.

Supplementary figures and tables

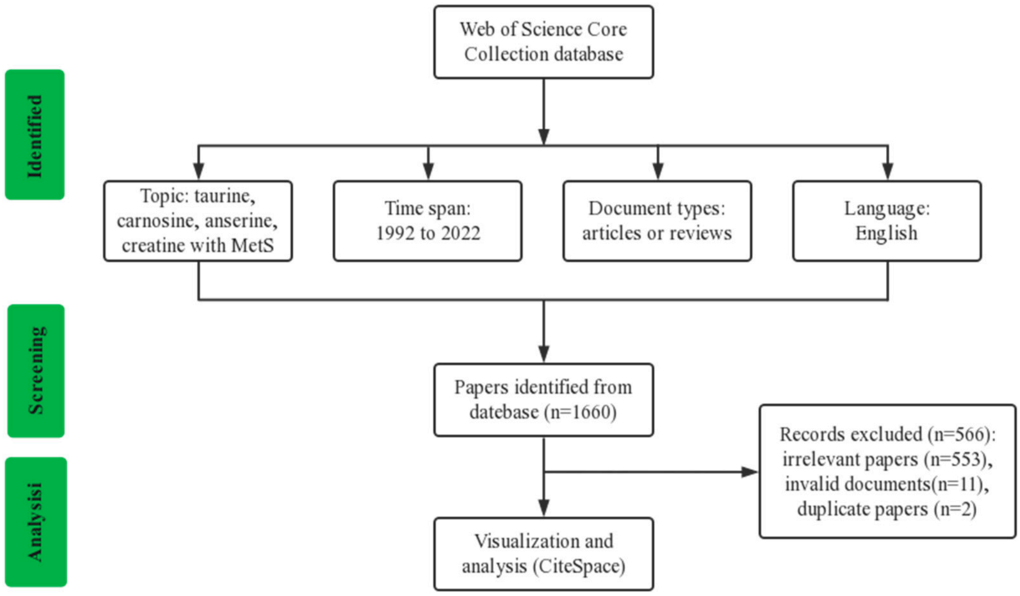

Figure S1. Flowchart of literature selection.

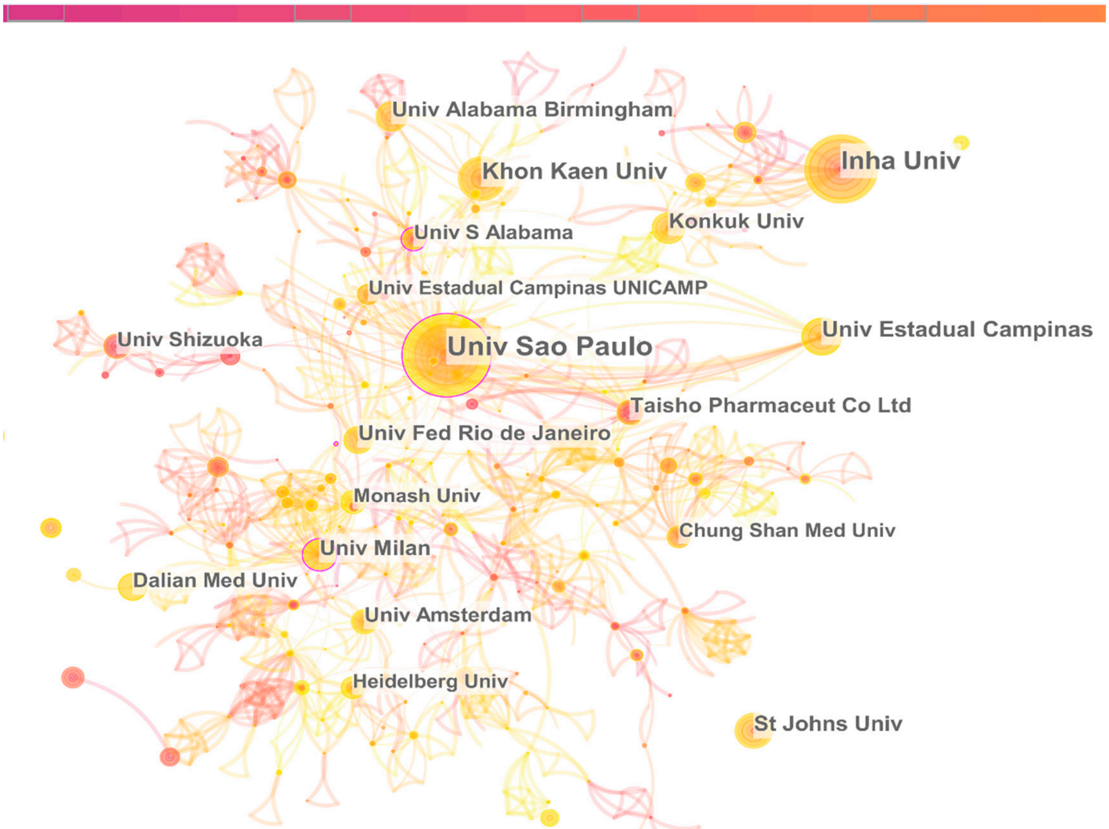

Figure S2. Collaboration network among research units at the institution level in the research field of taurine, creatine, carnosine, and anserine with metabolic syndrome. Here, research constituents of larger node size reveal that more papers have been published

from the unit. Nodes with purple ring indicate that they are central nodes in the network with the scale of centrality >0.1 (i.e., representing stronger influence and more intermediary connection in the knowledge networks). The thickness of purple ring is proportional to the centrality strength of nodes (i.e., the higher the centrality, the greater the influence of the research unit, the closer the connection with others). The number and thickness of links between nodes reflect their collaboration relationships. The color of nodes and links represent publication and first association time, respectively. Warmer color indicates a closer time.

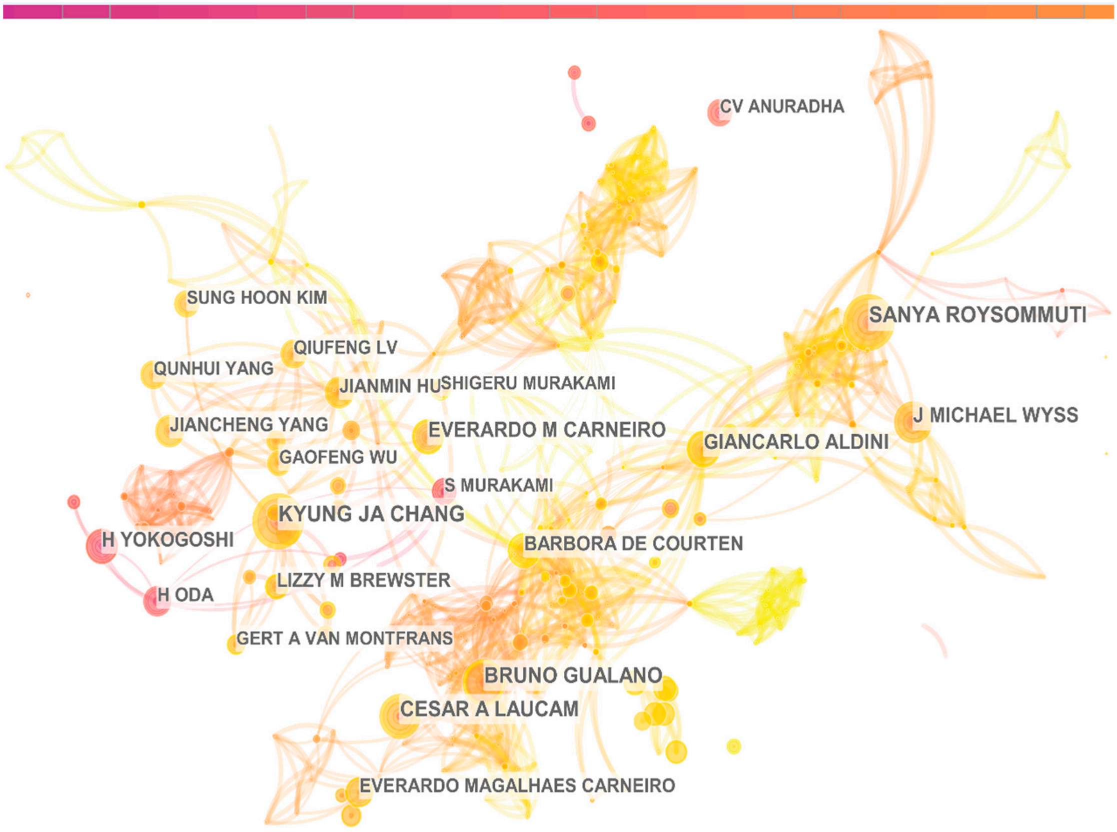

Figure S3. Collaboration network among research units at the authorship level in the research field of taurine, creatine, carnosine, and anserine with metabolic syndrome. Here, research constituents of larger node size reveal that more papers have been published from the unit. Nodes with purple ring indicate that they are central nodes in the network with the scale of centrality >0.1 (i.e., representing stronger influence and more intermediary connection in the knowledge networks). The thickness of purple ring is proportional to the centrality strength of nodes (i.e., the higher the centrality, the greater the influence of the research unit, the closer the connection with others). The number and thickness of links between nodes reflect their collaboration relationships. The color of nodes and links represent publication and first association time, respectively. Warmer color indicates a closer time.

Table S1. Top 12 most prolific journals related to taurine, carnosine, anserine and creatine with metabolic syndrome.

No.	Journal	No. of Publications (%)	IF [a]	JCR ® Category
**1**	Amino Acids	73 (6.7)	3.789	Biochemistry & Molecular Biology (Q3)
**2**	Aquaculture	17 (1.6)	5.135	Marine & Freshwater Biology (Q1)
**3**	Life Sciences	16 (1.5)	6.780	Pharmacology & Pharmacy (Q1)
**4**	PLoS One	16 (1.5)	3.752	Multidisciplinary Sciences (Q2)
**5**	Journal of Biomedical Science	15 (1.4)	12.771	Medicine, Research & Experimental (Q1)
**6**	Nutrients	13 (1.2)	6.706	Nutrition & Dietetics (Q1)
**7**	Journal of the International Society of Sports Nutrition	12 (1.1)	4.948	Nutrition & Dietetics (Q2)
**8**	Medicine and Science in Sports and Exercise	11 (1.0)	4.475	Science & Sports (Q1)
**9**	International Journal of Molecular Sciences	9 (0.8)	6.208	Biochemistry & Molecular Biology (Q1)
**10**	Journal of Agricultural and Food Chemistry	9 (0.8)	5.895	Chemistry, Applied (Q1)
**11**	Journal of Nutritional Biochemistry	9 (0.8)	6.117	Nutrition & Dietetics (Q1)
**12**	Nutrition Research	9 (0.8)	3.876	Nutrition & Dietetics (Q3)

Abbreviations: IF, impact factor; JCR, Journal Citation Reports.

[a]: Data from the 2022 edition of Journal Citation Reports was used.

Table S2. Top 10 cited references on taurine, carnosine, anserine and creatine with metabolic syndrome.

No.	Frequency [a]	Centrality [b]	Title	Author	Journal	Published year
**1**	30	0.37	Effects of carnosine supplementation on glucose metabolism: Pilot clinical trial.	de Courten B, et al.	Obesity	2016
**2**	28	0.03	The potential usefulness of taurine on diabetes mellitus and its complications.	Ito T, et al.	Amino Acids	2012
**3**	28	0.10	Physiology and pathophysiology of carnosine.	Boldyrev AA, et al.	Physiol Rev	2013
**4**	25	0.00	Role of taurine in the pathogenesis of obesity.	Murakami S	Mol Nutr Food Res	2015
**5**	24	0.05	Role of antioxidant activity of taurine in diabetes.	Schaffer SW, et al.	Can J Physiol Pharm	2009
**6**	24	0.02	Taurine supplementation modulates glucose homeostasis and islet function.	Carneiro EM, et al.	J Nutr Biochem	2009
**7**	21	0.00	The physiological and pathophysiological roles of taurine in adipose tissue in relation to obesity.	Murakami S	Life Sci	2017
**8**	20	0.00	Carnosine Attenuates the Development of both Type 2 Diabetes and Diabetic Nephropathy in BTBR ob/ob Mice.	Albrecht T, et al.	Sci Rep	2017
**9**	20	0.02	The role of taurine in diabetes and the development of diabetic complications.	Hansen SH	Diabetes-Metab Res	2001
**10**	19	0.02	The carbonyl scavenger carnosine ameliorates dyslipidaemia and renal function in Zucker obese rats.	Aldini G, et al.	J Cell Biochem	2011

[a]: Frequency indicates the number of publications citing the reference papers.

[b]: The higher the centrality, the greater the literature influence, the closer the connection with others.

Table S3. Top 10 countries/regions, institutions and authors of publications related to taurine, carnosine, anserine and creatine with metabolic syndrome.

No.	Countries /Regions	Frequency (%) [a]	Centrality [b]	Institution	Frequency (%)	Centrality	Authors	Frequency (%)	Centrality
1	USA	207 (18.9)	0.79	University of Sao Paulo	38 (3.5)	0.10	Kyung Ja Chang	21 (1.9)	<0.01
2	China	131 (12.0)	0.53	Inha University	33 (3.0)	0.01	Sanya Roysommuti	21 (1.9)	<0.01
3	Japan	101 (9.2)	0.25	Khon Kaen University	21 (1.9)	<0.01	Bruno Gualano	19 (1.7)	<0.01
4	Brazil	96 (8.8)	0.27	Universidade Estadual de Campinas	20 (1.8)	0.01	Cesar A Laucam	18 (1.7)	<0.01
5	South Korea	69 (6.3)	0.22	St. John's University	18 (1.7)	<0.01	Everardo M Carneiro	16 (1.5)	<0.01
6	Italy	45(4.1)	0.13	Taisho Pharmaceut Co Ltd	16 (1.5)	0.09	Giancarlo Aldini	16 (1.5)	<0.01
7	Canada	39 (3.6)	0.13	University of Milan	16 (1.5)	0.18	J Michael Wyss	16 (1.5)	<0.01
8	Australia	34 (3.1)	0.15	Konkuk University	16 (1.5)	0.01	Barbora De Courten	15 (1.4)	<0.01
9	England	32 (2.9)	0.10	Federal University of Rio de Janeiro	15 (1.4)	0.01	H Yokogoshi	15 (1.4)	<0.01
10	Netherlands	31 (2.8)	0.06	The University of Alabama at Birmingham	15 (1.4)	<0.01	Everardo Magalhaes Carneiro	13 (1.2)	<0.01

[a]: Frequency indicates the number of publications from each research unit (region/ institution/ author).

[b]: Centrality >0.1 indicates that the node is a central node, and the higher the centrality, the greater the influence of the country/region, institution and author, the closer the connection with others.
